# Long-term outcomes of percutaneous transluminal renal artery intervention: a retrospective study at a single center

**DOI:** 10.1186/s40885-024-00282-9

**Published:** 2024-08-01

**Authors:** In Sook Kang, Donghoon Choi, Young-Guk Ko, Dong-Ho Shin, Jung-Sun Kim, Byeong-Keuk Kim, Myeong-Ki Hong, Yangsoo Jang

**Affiliations:** 1 Division of Cardiology, Department of Internal Medicine, Ewha Womans University Mokdong Hospital, Ewha Womans University College of Medicine, Seoul, Republic of Korea; 2 Division of Cardiology, Department of Internal Medicine, Severance Cardiovascular Hospital, Yonsei University College of Medicine, Seoul, Republic of Korea; 3 Division of Cardiology, Department of Internal Medicine, CHA Gangnam Medical Center, CHA University, Seongnam, Republic of Korea

**Keywords:** Hypertension, Balloon angioplasty, Renal artery obstruction, Chronic kidney failure

## Abstract

**Background:**

The indications, benefits, and outcomes of percutaneous transluminal renal artery intervention (PTRI) remain controversial. The study purpose was to evaluate the long-term outcomes of PTRI in clinical practice.

**Methods:**

A retrospective review of 217 subjects (254 renal arteries; mean age, 59.8 years) who underwent PTRI based on medical database.

**Results:**

The most common cause of renal artery stenosis was atherosclerosis in 217 (85.4%), followed by Takayasu arteritis (TA) in 23 (9.1%), fibromuscular dysplasia in five (2.0%) and others in nine (3.5%). Mean follow-up duration was 5.7 ± 3.7 years. The first restenosis rate was 7.5% (*n* = 19; highest in TA: *n* = 9, 47.4%) and second restenosis occurred in six arteries (five TAs, one fibromuscular dysplasia). Follow-up blood pressure improved from 142.0/83.5 to 122.8/73.5 mmHg (*P* < 0.001). There was no change within 5 years’ follow-up in estimated glomerular filtration rate (*P* = 0.44), whereas TA changed from 69.8 ± 20.5 to 84.2 ± 17.9 mL/min/1.73 m² (*P* = 0.008). Progressive renal dysfunction was related to diabetes mellitus, chronic kidney disease, and peripheral artery obstructive disease on multivariate analysis with hazard ratios (95% confidence intervals) of 2.24 (1.21–4.17), 2.54 (1.33–4.84), and 3.93 (1.97–7.82), respectively.

**Conclusions:**

PTRI was associated with a blood pressure reduction. Despite a higher rate of restenosis, patients with TA showed significant improvement in estimated glomerular filtration rate. Diabetes mellitus, chronic kidney disease, and peripheral artery obstructive disease were related with progressive renal dysfunction after PTRI.

## Background

Renal artery stenosis (RAS) is a possibly reversible cause of renovascular hypertension and renal dysfunction [1]. The proportion of RAS is estimated at 1–5% in the hypertensive population [1, 2] and 6.8% in elderly persons [3]. The majority of RAS is caused by atherosclerosis [4]. Fibromuscular dysplasia (FMD) is known to be a distant second common cause and accounts for less than 10% of RAS cases [4, 5]. Less than 5% of RAS are associated with a variety of conditions, including thromboembolism, Takayasu arteritis (TA), renal artery dissection, and trauma-related conditions [4, 5]. The presence of peripheral artery disease (PAD) or coronary artery disease is a known clinical predictor of RAS with a high prevalence of 35% and 22%, respectively [6, 7]. Therefore, screening for RAS used to be matter of interest for interventional cardiologists. The number of renal artery interventions markedly increased between 1988 and 2006 in the United States [8].

However, the recent results from randomized clinical trials failed to show the benefit of percutaneous transluminal renal intervention (PTRI) over medical treatment [9–11]. Despite some limitations in these clinical trials [12–15], there was a decrease in the number of PTRI after 2006 in both atherosclerotic RAS (ARAS) and FMD [8]. The majority of recent clinical trials reported that ARAS is the most common cause of RAS, but other diseases are related RAS as well, such as FMD and TA [5]. Treatment strategies and prognosis differ between these diseases.

Further, there were no specific guidelines or treatment strategies regarding restenosis after PTRI. The restenosis rate of stainless steel renal artery stents was reportedly 17.4% at 9 months [10]. Several factors including vessel diameter, long stent length, female sex, and inadequate apposition were known to increase the risk of restenosis [16, 17]. Even though balloon angioplasty was considered as effective as other modalities in ARAS [16], there is no known best reintervention strategy for renal in-stent restenosis [18], Moreover, there are little available published data regarding treatment of renal restenosis except for ARAS.

The purpose of this study was to evaluate the long-term outcomes of PTRI and management patterns of restenosis after PTRI for various diseases and etiologies in clinical practice.

## Methods

### Ethics statement

This study was approved by the Institutional Review Board of the Severance Hospital Ethics Committee (No. 4-2014-1015). The requirement for informed consent was waived due to the retrospective nature of the study.

### Study population

**Fig. 1:**
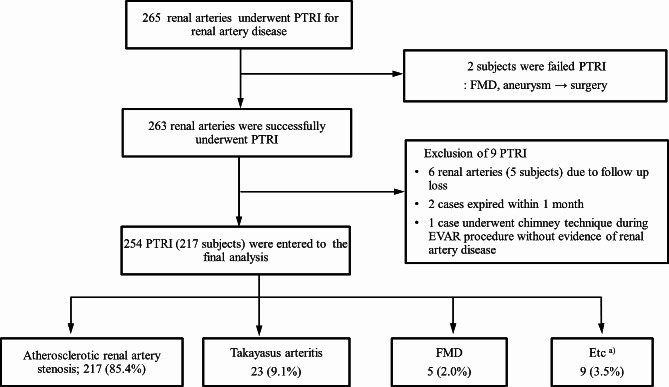
Flowchart of study population and proportion of each disease. PTRI, percutaneous transluminal renal intervention; FMD, fibromuscular dysplasia; EVAR, endovascular aneurysm repair. ^a)^Including three cases of aortic dissection involving the renal artery, two cases of bailout stenting after aorta intervention and abdominal vascular surgery, and four cases of renal infarction

This is a retrospective cohort study based on medical record from a single medical center. The study population included 228 subjects (265 renal arteries) who underwent PTRI in the cardiology department between 1994 and 2013. We only included those patients for whom medical records were available for at least 1 month after successful PTRI. Some pediatric patients with FMD or TA treated in the cardiology department were included. After the exclusion of 10 patients (11 arteries; two failed PTRI; six follow-up losses; two expired within 1 month; one case of no evidence of severe RAS), 254 arteries (217 patients) were analyzed in this study (Fig. 1). Patient demographics including renal function, underlying disease related to RAS, and renal invention details were evaluated.

### Percutaneous transluminal renal artery intervention

PTRI was performed for angiographic significant stenosis > 70% with a resting translesional mean pressure gradient > 10 mmHg or peak systolic pressure gradient > 20 mmHg [12, 19]. However, PTRI was performed without measuring the pressure gradient under the operators’ decisions in urgent cases, including acute renal artery dissection, evidence of renal thrombus, and acute renal infarction.

The common femoral artery was the usual vascular access site. The procedures were performed using a 6–7 F renal guiding catheter after systemic heparinization. A 0.014-inch guidewire was used to select the diseased renal artery, and balloon angioplasty was performed except in cases of direct stenting. Regarding stent placement, predilation was usually performed; however, direct stenting and/or adjuvant balloon angioplasty was performed as needed.

Technical success was defined as angiography showing residual stenosis of the target renal artery of < 30% at the end of the procedure after successful vascular access and an endovascular procedure [20].

### Study definitions and outcomes

Renal outcome was evaluated using estimated glomerular filtration rate (eGFR). To calculate eGFR, we used the simplified Modification of Diet in Renal Disease formula [21] for adults and the Bedside Schwartz formula for children [22]. Stages of chronic kidney disease (CKD) were classified by the Kidney Disease Outcomes Quality Initiative classification [23] according to the glomerular filtration rate (GFR): stage 1 (≥ 90 mL/min/1.73 m^2^), stage 2 (60–89 mL/min/1.73 m^2^), stage 3 (30–59 mL/min/1.73 m^2^), stage 4 (15–29 mL/min/1.73 m^2^), and stage 5 (< 15 mL/min/1.73 m^2^). In this study, we classified CKD based on the GFR 60 mL/min/1.73 m^2^, that is, stage 3 CKD.

We classified blood pressure (BP) outcomes according to the following definitions [18]: cure, restoration BP < 140/90 mmHg without antihypertensive drugs; improvement, systolic BP < 140 mmHg or diastolic BP < 90 mmHg with the same or reduced number of antihypertensive medications; and no effect, no change or could not satisfy the above criteria. BP outcomes were evaluated based on the last available medical record.

PAD is defined as atherosclerotic occlusion of blood vessels other than those of the heart and brain, usually involving the arteries of the lower extremities [24].

We counted major cardiovascular or renal events as major events [2]. Major cardiovascular events included death of cardiovascular or renal causes, stroke, myocardial infarction, and hospitalization due to heart failure. We also evaluated the need for permanent renal replacement therapy (RRT) and reducing renal function from a baseline of > 30% of eGFR as progressive renal dysfunction within 5 years after being discharged from successful PTRI.

### Statistical analysis

Baseline demographics were presented using the chi-square test for categorical variables and Student t-test or Fisher exact test for continuous variables. A paired t-test was used to compare baseline and follow-up data of BP, renal function, and number of antihypertensive drugs. The Cox proportional hazard model was used to evaluate the risk factors for progressive renal dysfunction and major events.

*P*-values of < 0.05 were considered statistically significant. The statistical analyses were performed using the IBM SPSS ver. 19.0 (IBM Corp).

## Results

### Baseline characteristics and restenosis

The most common etiology for PTRI was ARAS, followed by TA, urgent cases, and FMD. Bilateral PTRI was performed in 37 patients (74 lesions) due to bilateral disease, while four patients were treated due to RAS of a single functioning kidney (Fig. 1).

**Fig. 2:**
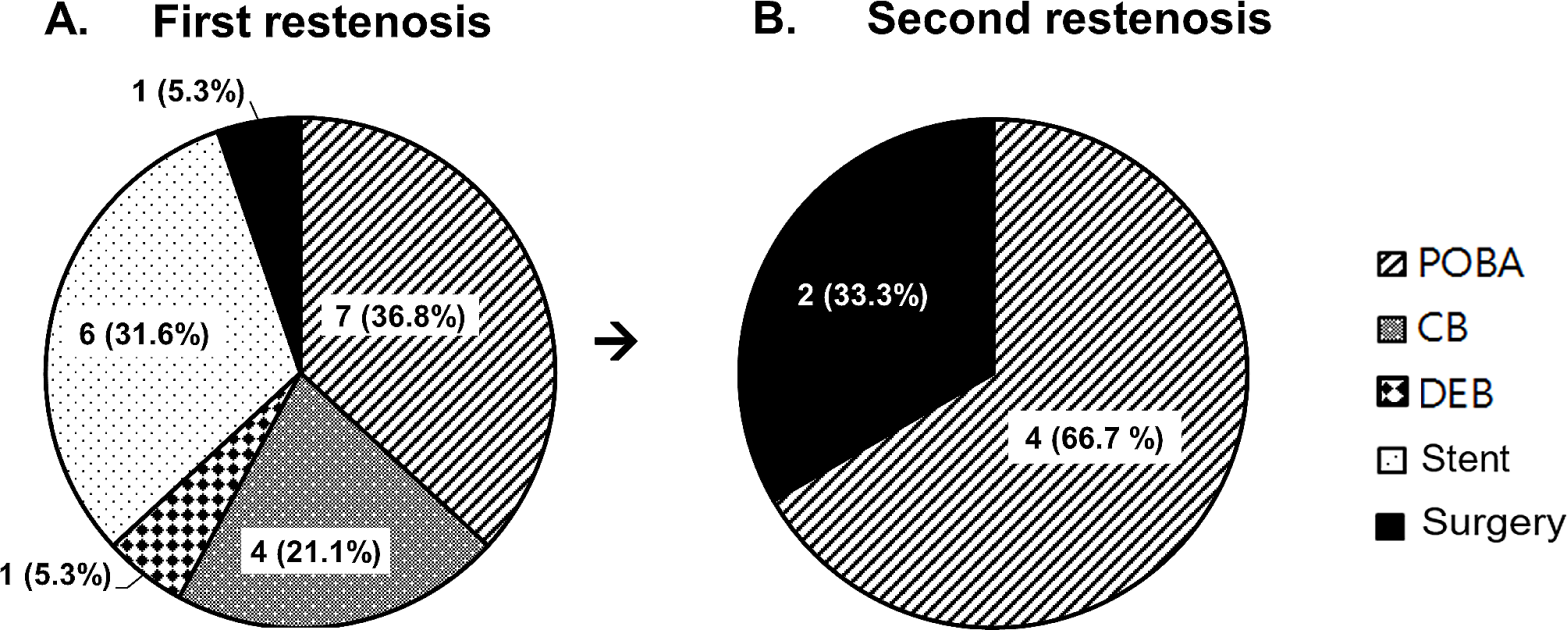
Treatment patterns for restenosis lesions after percutaneous transluminal renal intervention. (**A**) First restenosis (total of 19 lesions). (**B**) Second restenosis (total of six lesions) Numbers indicate number of lesions. POBA, plain old-type balloon angioplasty; CB, balloon angioplasty with cutting balloon; DEB, balloon angioplasty with drug-eluting balloon

The average age of all subjects was 59.8 ± 15.9 years, and those with ARAS tended to be older (*P* < 0.001) (Table 1). Diabetes mellitus (DM), coronary artery disease, heart failure, dyslipidemia, and CKD showed higher incidences in patients with ARAS than in those without ARAS. PAD did not differ between groups. Stent placement was conducted in 238 of 254 lesions (93.7%) and differed somewhat according to underlying diseases: ARAS, 209 out of 217 lesions (96.7%); TA, 21 out of 23 lesions (91.3%); urgent cases, seven out of nine lesions (77.8%); and FMD, one out of five lesions (20.0%). There were no differences in diameter and length of balloons and stents. The mean follow-up duration was 5.7 ± 3.7 years (median, 5.48 years; range, 1.2–246 months). Follow-up angiography was performed for 74 lesions, of which restenosis was evident in 19 (25.3% of lesions with follow-up angiography and 7.5% of total subjects) including nine lesions of patients with TA, seven lesions of patients with ARAS, and three lesions of patients with FMD. The average period until restenosis was 3.29 ± 3.17 years. Repeated restenosis occurred in six lesions in patients without ARAS (2.4% of the total subjects): five in patients with TA and one in a patient with FMD. Various modalities including plain, cutting, and drug-eluting balloon angioplasty, additional stent placement, and surgery were used to treat the cases of restenosis (Fig. 2). Plain old-type balloon angioplasty (POBA) was the most frequently used modality for restenotic lesions. Patients with TA showed a high incidence of restenosis including ≥ 3 times restenosis of two lesions that were successfully treated with POBA.

**Table 1:** Baseline characteristics and overall outcomes after PTRI

Characteristic	Total (*n* = 254)	ARAS (*n* = 217)	Non-ARAS (*n* = 37)	*P*-value
Average age	59.8 ± 15.9	63.1 ± 12.5	41.1 ± 20.1	< 0.001
Female sex	94 (37.0)	74 (34.1)	20 (54.1)	0.020
Body mass index (kg/m^2^)	23.6 ± 3.3	23.7 ± 3.2	23.1 ± 3.9	0.332
Diabetes mellitus	67 (26.4)	65 (30.0)	2 (5.4)	0.002
Peripheral artery disease	111 (43.7)	98 (45.2)	13 (35.1)	0.256
Coronary artery obstructive disease	181 (71.3)	164 (75.6)	17 (45.9)	< 0.001
Stroke	29 (11.4)	25 (11.5)	4 (10.8)	0.900
Atrial fibrillation	24 (9.6)	21 (9.8)	3 (8.3)	0.780
Congestive heart failure	37 (14.6)	35 (16.1)	2 (5.4)	0.087
Dyslipidemia	76 (29.9)	72 (33.2)	4 (10.8)	0.006
CRF (GFR < 60 mL/min/1.73 m^2^)	129 (50.8)	116 (53.5)	13 (35.1)	0.039
No. of antihypertensive medications				-
Baseline	2.6 ± 1.2	2.6 ± 1.1	2.8 ± 1.5	
Follow-up	2.4 ± 1.2^a)^	2.5 ± 1.2	2.1 ± 1.2^a)^	
PTRI type				
Stenting	238 (93.7)	209 (96.3)	29 (78.4)	< 0.001
Stent diameter (mm)	6.2 ± 0.9	6.2 ± 0.9	6.1 ± 0.8	0.588
Stent length (mm)	16.6 ± 3.9	16.3 ± 3.2	18.7 ± 7.1	0.083
POBA	16 (6.3)	8 (3.7)	8 (21.6)	< 0.001
Balloon diameter (mm)	4.9 ± 0.9	4.9 ± 0.9	4.9 ± 1.0	0.994
Balloon length (mm)	19.8 ± 4.1	19.8 ± 2.7	19.2 ± 9.0	0.721
First restenosis	19 (7.5)	7 (3.2)	12 (32.4)	< 0.001
Second restenosis	6 (2.4)	0	6 (16.2)	< 0.001
Progressive renal dysfunction	46 (18.6)	44 (20.8)	2 (5.7)	0.034
Major cardiovascular event	29 (11.4)	25 (11.5)	4 (10.8)	0.900
Five-year RRT	4 (1.6)	4 (1.8)	0	0.405
Major event	65 (26.3)	60 (28.3)	5 (14.3)	0.081

Data are presented as mean ± standard deviation or number (%)

PTRI, percutaneous transluminal renal intervention; ARAS, atherosclerotic renal artery stenosis; CRF, chronic renal failure; POBA, plain old-type balloon angioplasty; RRT, renal replacement therapy

^a)^*P* < 0.05 compared to baseline

### Urgent cases for PTRI

Nine patients (3.5%) underwent PTRI due to urgent conditions (Table 2). Four of nine patients presented with acute renal infarction due to thrombus or spontaneous dissection of the renal artery. Three of the four patients with acute renal infarction showed fair long-term outcomes after PTRI with maintained eGFR, and follow-up computed tomography (CT) angiography showed patency of the renal arteries. However, one patient who had a single functioning kidney with CKD stage 3 showed poor prognosis and required RRT just after the endovascular intervention. There were three cases of aortic dissection–related conditions that were successfully treated by stent placement. There were two cases of bailout stenting to fix flow limitations after aortic surgery and endovascular repair that also showed fair long-term clinical outcomes without restenosis.

**Table 2:** Summary of urgent cases for PTRI (*n* = 9)

Case no.	Sex	Age (yr)	Renal disease	Combined condition	PTRI	Clinical outcome
1	Male	58	Thrombosis with acute infarction	Atrial fibrillation	Stenting	Single functioning kidney Baseline GFR, 44.2 mL/min/1.73 m^2^ Progression to ESRD just after PTRI Sudden cardiac death at 75 days
2	Male	78	Thrombosis with acute infarction	Idiopathic	Thrombus aspiration, POBA	CKD stage 3 stationary (GFR, 56.7 to 41.1 mL/min/1.73 m^2^) Event-free for 6 years
3	Female	56	Thrombosis with acute infarction	SVT, MS	Urokinase infusion, POBA	Follow-up GFR, 92 to 79.2 mL/min/1.73 m^2^ Event-free for 7 years
4	Male	37	Infarction with spontaneous RA dissection	Idiopathic	Direct stenting	Follow-up GFR, 72.3 to 71.9 mL/min/1.73 m^2^ Event-free for 8 months
5	Female	72	RA dissection	Aortic dissection	Direct stenting	Aortic dissection involved the renal artery, which was treated by TEVAR with PTRI No events at 75 days’ follow-up
6	Male	35	RA dissection	Aortic dissection	Direct stenting	Event-free for 4 years
7	Male	31	Jailed flow^a)^	Aortic dissection	Stenting	Follow-up GFR, 50.5 to 57.5 mL/min/1.73 m^2^ Event-free for 11 years
8	Male	50	Jailed flow	Abdominal aortic surgery	Bailout stenting	Follow-up GFR, 17.5 to 67.9 mL/min/1.73 m^2^ Event-free for 7 months
9	Male	79	Jailed flow	AAA stent graft	Bailout stenting	Follow-up GFR, 51.9 to 54.4 mL/min/1.73 m^2^ Event-free for 4.3 years

PTRI, percutaneous transluminal renal intervention; ESRD, end-stage renal disease; POBA, plain old-type balloon angioplasty; CKD, chronic kidney disease; SVT, supraventricular tachycardia; MS, mitral stenosis; RA, renal artery; TEVAR, thoracic endovascular aneurysm repair; AAA, abdominal aortic aneurysm

^a)^External compression by false lumen of aortic dissection

### Outcomes after PTRI

**Fig. 3:**
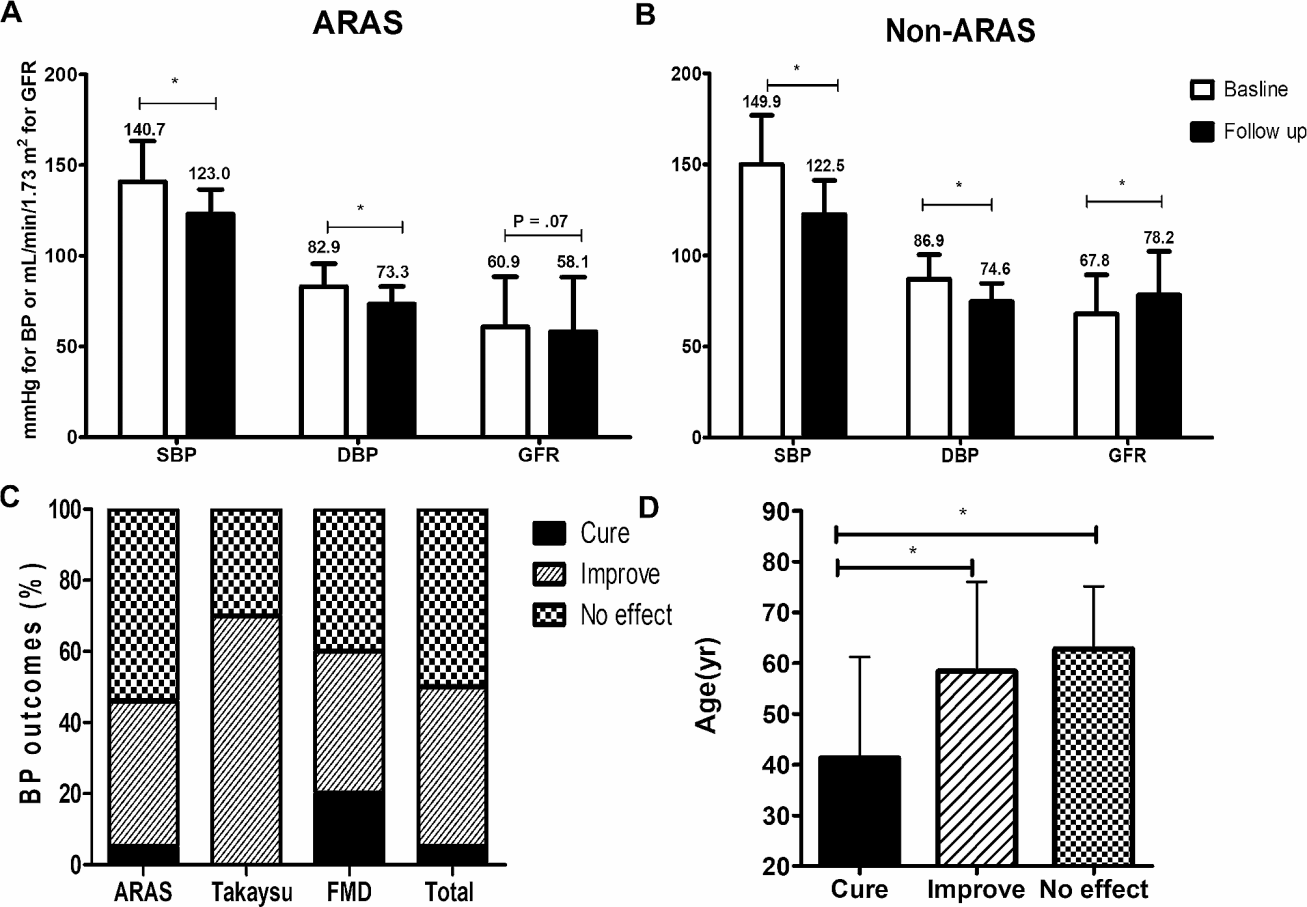
Blood pressure and glomerular filtration rate (GFR) outcomes. (**A, B**) Outcomes of atherosclerotic renal artery stenosis (ARAS) and non-ARAS. Blood pressure outcomes for follow-up periods and GFR within 5 years. (**C, D**) Blood pressure outcomes classified into cure, improvement, and no effect groups. BP, blood pressure; SBP, systolic blood pressure; DBP, diastolic blood pressure; FMD, fibromuscular dysplasia. ^*^P < 0.001

After PTRI, average systolic BP decreased from 142.1 ± 23.4 to 122.9 ± 14.3 mmHg and average diastolic BP decreased from 83.5 ± 12.9 to 73.5 ± 9.8 mmHg (*P* < 0.001 for both) (Fig. 3). The number of antihypertensive medications did not change in patients with ARAS, while those without ARAS showed a significant reduction in medications (*P* = 0.090 vs. *P* = 0.004) (Table 1).

Figure 3C shows the BP responses of cure, improvement, or no effect. Only 50% of the lesions were classified as cure or improvement; the other half of the lesions had no effect. There were some differences in response according to diseases. Figure 3D shows the difference in the average ages according to BP response. Patients in the cure group were the youngest (38.7 ± 21.0 years), while those in the no effect group were the oldest (62.78 ± 12.3 years). The status of “BP cure” was related to age after adjustment for DM, baseline systolic BP, GFR, and ARAS versus non-ARAS (*P* = 0.002), while “BP improve” was related to baseline systolic BP (*P* < 0.001) and ARAS versus non-ARAS (*P* = 0.004) but not age, DM, or GFR.

**Fig. 4:**
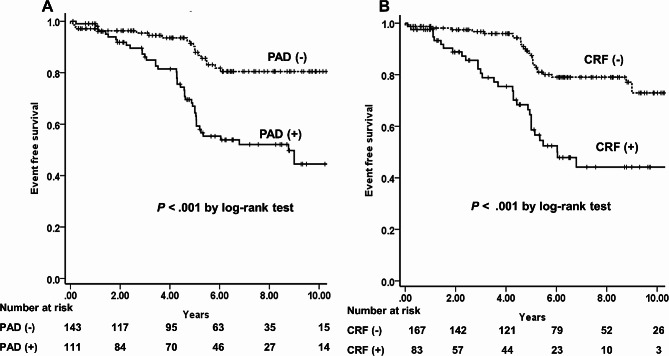
Kaplan-Meier survival curve for major cardiovascular event according to (**A**) peripheral artery disease (PAD) and (**B**) chronic renal failure (CRF).

Within 5 years’ follow-up, GFR improved in patients without ARAS from 67.8 ± 21.5 to 78.2 ± 24.0 mL/min/1.73 m^2^ (*P* = 0.019) but not in patients with ARAS (*P* = 0.07) (Fig. 3A, B). Progressive renal dysfunction was more frequent in ARAS than non-ARAS. Table 3 shows DM, baseline chronic renal failure, and PAD related with increasing hazard ratio (HR) on multivariate analysis. PTRI for bilateral RAS or a single functioning kidney failed to decrease HR for progressive renal dysfunction. Permanent RRT was newly required in four lesions and all subjects had ARAS (one subject with two lesions, bilateral RAS, and baseline CKD stage 4; and two subjects with CKD stages 3 and 4). Major events were insignificantly more frequent in patients with ARAS (*P* = 0.08). Baseline CKD and PAD were related with an increasing risk of major events on univariate and multivariate analysis (HR [95% confidence interval], 1.92 [1.14–3.24] and 2.80 [1.63–3.24], respectively) but DM showed significance on univariate analysis only. Figure 4 shows the long-term event-free survival for major events according to baseline CKD and PAD.

**Table 3:** Progressive renal dysfunction

Variable	Univariate analysis			Multivariate analysis		
	HR	95% CI	*P*-value	HR	95% CI	*P*-value
Age (yr) vs. ≤50 yr			0.063			0.841
> 50–60	7.76	1.73–34.85	0.008	2.01	0.36–11.20	0.425
> 60–70	6.79	1.53–30.14	0.012	2.21	0.40–12.16	0.362
> 70	7.01	1.53–32.07	0.012	2.05	0.36–11.61	0.417
Diabetes mellitus	2.68	1.49–4.81	0.001	2.24	1.21–4.17	0.011
CRF (GFR < 60 mL/min/1.73 m^2^)	3.05	1.64–5.68	< 0.001	2.54	1.33–4.84	0.005
Peripheral artery disease	4.00	2.07–7.74	< 0.001	3.93	1.97–7.82	< 0.001
Coronary artery obstructive disease	3.07	1.29–7.32	0.012	1.40	0.55–3.53	0.479
Current smoker	1.19	0.63–2.22	0.598	-	-	-
Bilateral or solitary kidney	0.91	0.50–1.68	0.767	-	-	-
Female sex	0.72	0.52–0.99	0.049	0.94	0.47–1.88	0.853
ARAS vs. non-ARAS	5.23	1.26–21.77	0.023	2.76	0.54–14.16	0.223
Stenting vs. POBA	0.81	0.20–3.33	0.766	-	-	-
Restenosis	0.49	0.15–1.60	0.236	-	-	-
BP outcome vs. no effect			0.350	-	-	-
Improvement	0.68	0.37–1.23	0.200			
Cure	0.43	0.06–3.17	0.408			

HR, hazard ratio; CI, confidence interval; CRF, chronic renal failure; GFR, glomerular infiltration rate; ARAS, atherosclerotic renal artery stenosis; POBA, plain old-type balloon angioplasty; BP, blood pressure

## Discussion

This study showed differences in outcomes after PTRI according to underlying etiology. TA and FMD were related to a higher rate of restenosis than ARAS. Although repeated POBA was frequently used in many cases, it was difficult to determine which modality was better for restenosis management. Nevertheless, repeated interventions with various modalities were feasible in the most cases. Consequently, despite the higher restenosis rate, patients with TA and FMD showed significantly improved eGFR in addition to improved BP and a reduced number of antihypertensive medications. Additionally, renal thrombosis-related acute renal infarction could be managed with thrombus aspiration or thrombolytic infusion and balloon angioplasty. Our data also showed favorable short- and long-term outcomes of direct stenting for renal artery dissection and bailout stenting.

Similar to those of previous multicenter trials [25, 26], our data showed some benefits of PTRI for controlling BP and no significant improvement in eGFR but maintained baseline eGFR in patients with ARAS. Contrary to patients with ARAS, in whom no cases of second or more restenosis were evident in this study, patients with TA showed a higher restenosis rate of 39% (*n* = 9), which was comparable to the 33.3% restenosis rate in another study [27]. Additionally, our data showed a 17% rate of two or more restenosis episodes. Despite the repeated restenosis, second and third interventions were feasible in most cases. In particular, repeated POBA was performed successfully and showed no alternation in clinical outcomes. However, totally occluded lesions were related technical failures and required surgery.

Patients with FMD presented with a similar disease progress and outcomes to those with TA after PTRI; high restenosis rate and improvement of BP and eGFR. However, there were only five lesions in patients with FMD, which made it difficult to draw statistical comparisons. Although the prevalence of FMD is reportedly 0.4% in the general population and 10% in patients with renovascular hypertension, it is more common in the Caucasian population and rare in the Asian population [28, 29], while TA is dominant in Asian women [30, 31].

Normal decreases in GFR with aging should be considered to assess the renal outcomes in terms of changes in eGFR. Data from mass health screening tests in the Republic of Korea showed that eGFR decreased from 72.7 to 68 mL/min/1.73 m^2^ in men and from 71.1 to 64.5 mL/min/1.73 m^2^ in women aged 65 to 75 years [32]. Hence, it is not easy to discriminate whether the decrease seen in this study is due to the natural course of aging or comes from renal artery disease or another cause since we have no control groups. We assessed eGFR outcomes within 5 years to minimize the aging-related effect. Our data showed a similar rate of progressive renal dysfunction in patients with ARAS as 21.0% compared to the 18.9% reported in the Cardiovascular Outcomes in Renal Atherosclerotic Lesions (CORAL) study [2] and a slightly lower rate of major cardiovascular and renal events of 28.6% in patients with ARAS versus 35.8% in the CORAL study.

Although there are limited available data regarding the risk factors related to poor clinical outcomes after PTRI, age was reported as one risk factor, and the proportion of improvement in eGFR after PTRI was smaller and eGFR aggravation occurred more frequently in patients > 65 years [33, 34]. In this study, there may be two reasons for a lack of achieving eGFR improvement in patients with ARAS: older average age of patients with ARAS than those without ARAS; and ARAS may be related to chronic conditions including chronic atherosclerosis, long-term hypertension, and renal parenchymal changes unlike FMD or renal artery dissection, which are more closely related to renal artery disease. For that reason, the improvement in eGFR was minimal or baseline values were maintained after correction for RAS in the ARAS group. Furthermore, BP outcomes of young patients with FMD and normal renal function showed a higher cure rate, and it was reported that the cure rate of hypertension decreased with age similar to ARAS in FMD [29]. In the current study, BP cure was also related to age after adjustment for covariates. However, progressive renal dysfunction was correlated with age on univariate analysis but not multivariate analysis. DM, baseline CKD, and PAD were more important risk factors for progressive renal dysfunction in this study.

Stent placement in patients with ARAS was related to improved durability and good results [25, 35], and the optimal endovascular treatment for FMD is balloon angioplasty [5]. It remains controversial whether stent placement or PTRI is optimal for patients with TA [27, 31, 36]. Additionally, our data showed that urgent conditions including acute renal flow limitation were amenable to immediate endovascular treatment (Table 2). Direct stenting showed good long-term results on renal artery dissection resulting from aortic dissection, and POBA with thrombus aspiration/thrombolytic infusion was also effective in the cases of renal artery thrombosis.

This study has some limitations. This was a single-center retrospective cohort study. Even though we had access to long-term follow-up data in the patients’ medical records, it is limited compared to a well-designed prospective study. Our study populations were usually followed up based on outpatient clinics and our patients underwent regular BP check-ups and renal function tests. However, regarding imaging studies for the evaluation of renal flow patency, renal angiography, Doppler ultrasonography, and CT scans were not routinely performed in all subjects; rather, was performed according to physician discretion. All nine patients shown in Table 2 underwent regular CT angiography, while only 75 of total 245 subjects (30.6%) underwent follow-up renal angiography. Therefore, there is a chance that we underestimated the restenosis rate in asymptomatic subjects in whom eGFR was maintained and BP was well controlled.

## Conclusions

Our results showed that PTRI was associated with reduced BP and a decreased number of antihypertensive medications rather than improved eGFR. Despite a higher restenosis rate, patients with TA showed significantly improved eGFR. DM, CKD, and PAD were related to progressive renal dysfunction after PTRI. Additionally, chronic renal failure and PAD were related to an increased incidence of major events after PTRI.

## Data Availability

Not applicable.

## Abbreviations

ARAS: Atherosclerotic renal artery stenosis
BP: Blood pressure
CB: Balloon angioplasty with cutting balloon
CKD: Chronic kidney disease
CORAL: Cardiovascular Outcomes in Renal Atherosclerotic Lesions
CT: Computed tomography
DM: Diabetes mellitus
eGFR: Estimated glomerular filtration rate
EVAR: Endovascular aneurysm repair
FMD: Fibromuscular dysplasia
GFR: Glomerular filtration rate
HR: Hazard ratio
PAD: Peripheral artery disease
POBA: Plain old-type balloon angioplasty
PTRI: Percutaneous transluminal renal intervention
RAS: Renal artery stenosis
RRT: Renal replacement therapy
TA: Takayasu arteritis
TEVAR: Thoracic endovascular aneurysm repair
